# Surgical cytoreduction and hyperthermic intrathoracic chemotherapy for thymic tumours with pleural spread is effective on survival: results from the multicentre German hyperthermic intrathoracic chemotherapy study

**DOI:** 10.1093/icvts/ivad032

**Published:** 2023-02-10

**Authors:** Michael Ried, Mohamed Hassan, Bernward Passlick, Severin Schmid, Till Markowiak, Karolina Müller, Gunnar Huppertz, Michael Koller, Hauke Winter, Laura V Klotz, Rudolf Hatz, Julia Kovács, Julia Zimmermann, Hans-Stefan Hofmann, Martin E Eichhorn

**Affiliations:** Department of Thoracic Surgery, University Hospital Regensburg, Regensburg, Germany; Department of Thoracic Surgery, Medical Center—University of Freiburg, Freiburg, Germany; Faculty of Medicine, University of Freiburg, Freiburg, Germany; Department of Thoracic Surgery, Medical Center—University of Freiburg, Freiburg, Germany; Faculty of Medicine, University of Freiburg, Freiburg, Germany; Department of Thoracic Surgery, Medical Center—University of Freiburg, Freiburg, Germany; Faculty of Medicine, University of Freiburg, Freiburg, Germany; Department of Thoracic Surgery, University Hospital Regensburg, Regensburg, Germany; Center for Clinical Studies, University Hospital Regensburg, Regensburg, Germany; Center for Clinical Studies, University Hospital Regensburg, Regensburg, Germany; Center for Clinical Studies, University Hospital Regensburg, Regensburg, Germany; Department of Thoracic Surgery, Thoraxklinik, University Hospital Heidelberg, Heidelberg, Germany; Member of the German Center for Lung Research (DZL), Translational Lung Research Center (TLRC) Heidelberg, Heidelberg, Germany; Department of Thoracic Surgery, Thoraxklinik, University Hospital Heidelberg, Heidelberg, Germany; Member of the German Center for Lung Research (DZL), Translational Lung Research Center (TLRC) Heidelberg, Heidelberg, Germany; Department of Thoracic Surgery, Ludwig-Maximilians-University of Munich, Munich, Germany; Asklepios Lung Clinic Gauting, Gauting, Germany; Department of Thoracic Surgery, Ludwig-Maximilians-University of Munich, Munich, Germany; Asklepios Lung Clinic Gauting, Gauting, Germany; Department of Thoracic Surgery, Ludwig-Maximilians-University of Munich, Munich, Germany; Asklepios Lung Clinic Gauting, Gauting, Germany; Department of Thoracic Surgery, University Hospital Regensburg, Regensburg, Germany; Department of Thoracic Surgery, Hospital Barmherzige Brüder Regensburg, Regensburg, Germany; Department of Thoracic Surgery, Thoraxklinik, University Hospital Heidelberg, Heidelberg, Germany; Member of the German Center for Lung Research (DZL), Translational Lung Research Center (TLRC) Heidelberg, Heidelberg, Germany

**Keywords:** Hyperthermic intrathoracic chemotherapy, Hyperthermic intrathoracic chemotherapy, Thymic tumour, Thymic carcinoma, Cytoreductive surgery

## Abstract

**OBJECTIVES:**

Cytoreductive surgery and hyperthermic intrathoracic chemotherapy (HITOC) is effective on survival for patients with pleural metastatic thymic tumours.

**METHODS:**

Multicentre, retrospective analysis of patients with stage IVa thymic tumours treated with surgical resection and HITOC. Primary end point was overall survival, secondary end points were recurrence-/progression-free survival and morbidity/mortality.

**RESULTS:**

A total of *n* = 58 patients (thymoma, *n* = 42; thymic carcinoma, *n* = 15; atypical carcinoid of the thymus, *n* = 1) were included, who had primary pleural metastases (*n* = 50; 86%) or pleural recurrence (*n* = 8; 14%). Lung-preserving resection (*n* = 56; 97%) was the preferred approach. Macroscopically complete tumour resection was achieved in *n* = 49 patients (85%). HITOC was performed with cisplatin alone (*n* = 38; 66%) or in combination with doxorubicin (*n* = 20; 34%). Almost half of the patients (*n* = 28; 48%) received high-dose cisplatin > 125 mg/m^2^ body surface area. Surgical revision was required in 8 (14%) patients. In-hospital mortality rate was 2%. During follow-up, tumour recurrence/progression was evident in *n* = 31 (53%) patients. Median follow-up time was 59 months. The 1-, 3- and 5-year survival rates were 95%, 83% and 77%, respectively. Recurrence/progression-free survival rates were 89%, 54% and 44%, respectively. Patients with thymoma had significantly better survival compared to patients with thymic carcinoma (*P*-value ≤0.001).

**CONCLUSIONS:**

Promising survival rates in patients with pleural metastatic stage IVa in thymoma (94%) and even in thymic carcinoma (41%) were achieved. Surgical resection and HITOC is safe and effective for treatment of patients with pleural metastatic thymic tumours stage IVa.

**Figure:**
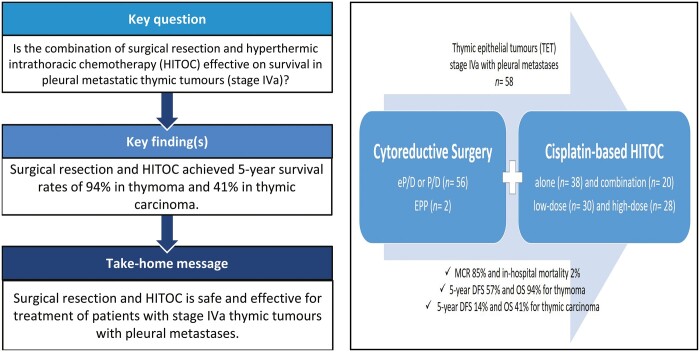

## INTRODUCTION

The surgical treatment of patients with advanced, pleural metastatic thymic epithelial tumours (TET) in stage IVa has been the subject of numerous studies for years. Due to the rarity of the tumour, however, these studies typically include only small numbers of patients [1–5]. The European Society for Medical Oncology guidelines recommend multimodal therapy including surgery for patients in stage IVa (pleural involvement), for whom complete resection is deemed feasible [6]. An analysis of the ESTS database has already confirmed that even in stage IVa, surgical resection of the pleural tumour components leads to good local tumour control and can consequently also improve the overall survival (OS) of patients [7]. Despite encouraging survival rates after pleural tumour resection within a multimodal approach, pleural recurrences limit patient survival [8].

Similar to the malignant pleural mesothelioma (MPM), only a macroscopically complete tumour resection (R0/R1) seems technically achievable due to the mostly diffuse tumour spread on the pleural surface. For technical reasons, a safety margin cannot be maintained, so that a microscopic residual tumour must be assumed. Pleural relapses are to be referred to microscopic invisible implants or TET cell spread at the moment of surgical removal. For years, additional intracavitary therapy approaches have been investigated to improve local tumour control, with hyperthermic intrathoracic chemotherapy (HITOC) being increasingly performed [9–12]. In addition to MPM, TET with pleural involvement are suitable for a combined surgical approach including HITOC [13, 14]. However, reliable data are lacking for this particular therapy, as only feasibility studies and small retrospective studies have been published so far.

The aim of this study is to investigate the combination of cytoreductive surgery (CRS) and HITOC in a multicentric cohort of patients with pleural metastatic TET stage IVa, especially with regard to disease-free and OS as well as therapy-associated complications and mortality. Our study hypothesis states that the combination of CRS + HITOC is a safe and for survival effective surgical treatment for patients with TET stage IVa (pleural).

## PATIENTS AND METHODS

### Study design

We retrospectively analysed a subgroup of patients with TET and pleural spread (stage IVa), which were collected in the ‘German HITOC study’, a multicentre, retrospective study funded by the Deutsche Forschungsgemeinschaft—DFG, German Research Foundation (GZ: RI 2905/3–1) [13, 15]. The trial is registered in the German Registry of Clinical Studies (DRKS-ID: DRKS00015012).

### Ethical statement

This study was conducted according to the guidelines of the Declaration of Helsinki, and approved by the Ethics Committee of the University of Regensburg (reference number: 18-1119-104) and of the ethics committees of the participating centres.

### Setting

All patients underwent elective surgery in 1 of the 4 participating high-volume departments for thoracic surgery in Germany: Regensburg, Munich, Heidelberg, Freiburg. Patient consent was waived due to the retrospective design of the study and collection of routine clinical data. The existing database was supplemented by an additional collection of clinical and follow-up data, which were updated until November 2021.

### Participants

The study protocol of the entire study specified the inclusion and exclusion criteria for the German HITOC study [13, 15]. In this analysis, a subgroup of patients with TET stage IVa due to pleural metastases, who underwent CRS and cisplatin-based HITOC within 1 surgery were included. Patients without HITOC or without radical surgical resection were excluded. Both primary stage IVa as well as pleural recurrence of TET were included. The choice of chemotherapeutic agents and also the dosage of chemotherapeutic agents for HITOC was determined by each participating centre according to its own experience and protocols.

### Definition of variables

Patients were classified according to the 8th edition of the TNM classification of malignant tumours [16]. All patients had preoperative stage IVa due to TET with pleural spread, but 2 of them changed in postoperative stage IVb with additional histological positive nodal status. TET were classified according to the valid World Health Organization (WHO) classification into histological subtypes, where WHO types A–B3 are defined as thymoma [17]. CRS was performed as pleurectomy/decortication (P/D), extended P/D (eP/D) or extrapleural pneumonectomy (EPP). eP/D was defined as P/D with additional partial or complete resection of the diaphragm and/or pericardium. The concentrations of cisplatin and doxorubicin were documented in mg/m^2^ body surface area (BSA). Moreover, concentration for cisplatin was categorized in low (≤ 125 mg/m^2^ BSA) vs high (> 125 mg/m^2^ BSA) dose. Additive chemotherapy was defined as either induction chemotherapy and/or adjuvant chemotherapy. Postoperative complications were documented according to the Clavien–Dindo classification [18]. The beginning of primary therapy was defined as date of CRS + HITOC if no induction chemotherapy was applied, or if induction chemotherapy was applied, as the date of 1st application of induction chemotherapy. OS was defined as time from beginning of the primary therapy until death from any cause. Recurrence and progression were defined as documented intrathoracic, ipsilateral and/or contralateral tumour detection by cytology/histology and/or imaging. Recurrence and progression-free survival (RFS/PFS) was defined as time from beginning of the primary therapy until 1st objective tumour recurrence/progression or death from any cause, which ever occurred first [19].

### Endpoints

Primary endpoint was to analyse the OS. Secondary endpoints included disease-free survival as well as postoperative morbidity and mortality.

### Statistical analysis

All statistical analyses were conducted using the software package SPSS (version 26 or higher). Descriptive analyses were done for demographic and baseline characteristics using frequency (n), percentage (%), mean (*m*), standard deviation (SD), median (med), interquartile range (IQR) and range (min/max) depending on scale level and distribution of the parameters. Median follow-up time was calculated using the reversed Kaplan–Meier method. OS and RFS/PFS were analysed by the Kaplan–Meier estimator. The estimates for the probability of surviving were presented for the specific time points: 1-year, 3-year and 5-year survival rate (estimate). Moreover, the median survival time with 95% CI were presented when possible.

In univariable analyses (log-rank tests), OS and PFS/RFS were compared between cisplatin dosage, chemotherapeutical agent, resection status, histological subtype, postoperative renal insufficiency (acute kidney injury) and additive chemotherapy. The estimates for the probability of surviving were presented graphically in a Kaplan–Meier survival curve as well as for the specific time points: 1, 3 and 5 years.

Additionally, multivariable Cox proportional hazard regression models (enter method) were calculated. For the end point PFS/RFS, the clinical parameters cisplatin dosage, chemotherapeutical agent, resection status, histological subtype and additive chemotherapy as well as the covariate time between initial diagnosis and beginning of primary therapy were included. Regarding the end point OS, a limitation of number of variables was necessary due to the low number of events (*n* = 13). With an acceptable ratio of 5–9 events per predictor variable [20], only the variables histological subtype and additive chemotherapy, with *P*-values <0.1 in univariable analyses, were included. Hazard ratios (HRs) with corresponding 95% CI were presented. The level of significance was set at *P*_two-sided_ ≤ 0.050 for all tests.

## RESULTS

### Patient characteristics

The results of a subgroup (*n* = 58) from the entire patient population (*n* = 350) of the multicentre German HITOC study are presented. The mean age was 49.5 years (13.0 SD) with slightly more male patients (62.1%; Table 1). The mean Karnofsky index was 89.1% (8.0% SD) and 94.8% of patients were Eastern Cooperative Oncology Group ≤ 1. With 96.6% (*n* = 56), nearly all patients had TET in stage IVa, only 3.4% (*n* = 2) were postoperatively classified as stage IVb due to additional positive lymph nodes. The majority of patients were 1st diagnosed with a TET with pleural spread (86.2%), but also patients with pleural recurrences (13.8%) were included. Thymomas (*n* = 42) were classified into histological subtypes: AB (*n* = 1; 2.4%), B1 (*n* = 8; 19.0%), B2 (*n* = 24; 57.1%) and B3 (*n* = 9; 21.4%). A total of 15 (25.9%) patients had thymic carcinoma and 1 patient (1.7%) was diagnosed with an atypical carcinoid of the thymus.

**Table 1:** Patient characteristics and clinical baseline data

Study population, *n* = 58	
Gender, *n* (%)	
Female	22 (37.9)
Male	36 (61.1)
Age (years)	49.5 (13.0)
BSA (m^2^), mean (SD)	1.9 (0.2)
BMI (kg/m²), mean (SD)	25.4 (4.3)
Karnofsky index, *n* (%)	
70%	3 (5.2)
80%	11 (19.0)
90%	29 (50.0)
100%	12 (20.7)
Missing	3 (5.2)
ECOG status, *n* (%)	
0	41 (70.7)
1	14 (24.1)
Missing	3 (5.2)
Pleural tumour manifestation, *n* (%)	
Primary tumour	50 (86.2)
Recurrence	8 (13.8)
Side of tumour, *n* (%)	
Left	29 (50.0)
Right	29 (50.0)
Histology,^a^*n* (%)	
Thymoma	42 (72.4)
Thymic carcinoma	15 (25.9)
Atypical carcinoid of the thymus	1 (1.7)
CRS	
Surgical procedure, *n* (%)	
P/D	12 (20.7)
eP/D	44 (75.9)
EPP	2 (3.4)
Resection of, *n* (%) (>100%)	
Diaphragm	37 (63.8)
Pericardium	27 (46.6)
Chest wall	3 (5.2)
Lung (wedge/atypical)	24 (41.4)
Alloplastic reconstruction/replacement of, *n* (%)	
Diaphragm	19 (32.8)
Pericardium	22 (37.9)
Resection status, *n* (%)	
R0/1	49 (84.5)
R2	9 (15.5)
Blood loss,^b^ median (IQR)	575 (100–950)
Transfusion PC#,^b^ median (IQR)	0 (0–0)
Transfusion FPP#,^b^ median (IQR)	2 (0–4)
Transfusion PRBC#,^b^ median (IQR)	1 (0–2)
Intraoperative complication, *n* (%)	
No	57 (98.3)
Yes	1 (1.7)
HITOC	
Duration of HITOC, median (IQR) (min)	60 (60–60)
Temperature perfusate (°C)	
Minimum, median (IQR) (*n* = 56)	42.0 (41.5–42.0)
Maximum (median, IQR) (*n* = 56)	42.0 (42.0–42.5)
Pump flow (ml/min)	
Minimum, median (IQR) (*n* = 56)	1200 (1000–1375)
Maximum, median (IQR) (*n* = 56)	1200 (1000–1500)
Perfusion volume (ml), median (IQR) (*n* = 58)	5000 (4000–5000)
Chemotherapeutic agents, *n* (%)	
Cisplatin	38 (65.5)
Cisplatin + doxorubicin	20 (34.5)
Total dosages of chemotherapeutic agents (mg/m^2^ BSA), median (IQR)	
Cisplatin (*n* = 58)	120 (100.0–175.0)
Doxorubicin (*n* = 20)	35.3 (32.3–38.1)
Cisplatin dosage (mg/m^2^ BSA), *n* (%)	
Low dose ≤125	30 (51.7)
High dose >125	28 (48.3)
Complications during HITOC, *n* (%)	
No	58 (100)
Yes	0 (0)

a WHO histological classification [14].

b Blood loss and transfusion was recorded in *n* = 24 patients (41.4%).

BMI: body mass index; BSA: body surface area; CRS: cytoreductive surgery; ECOG: Eastern Cooperative Oncology Group; eP/D: extended pleurectomy/decortication; EPP: extrapleural pneumonectomy; HITOC: hyperthermic intrathoracic chemotherapy; IQR: interquartile range; P/D: pleurectomy/decortication; SD: standard deviation; PC: platelet concentrate; PRBC: packed red blood cells; FFP: fresh frozen plasma; OR: operation room.

### Surgical approach

Lung-sparing P/D or eP/D were the preferred resection techniques in *n* = 56 (96.6%) patients and only 2 patients received EPP (Table 1). When necessary, alloplastic reconstruction of the diaphragm (*n* = 19; 32.8%) or pericardium (*n* = 22; 37.9%) was performed. A macroscopically complete resection (R0/R1) was achieved in *n* = 49 (84.5%) patients. After completion of the CRS, subsequent HITOC was performed in the same session for 60 min at a temperature of ∼42°C and a flow rate of 1200 ml/min. Intrathoracic chemotherapy consisted of cisplatin, either as a single dose (*n* = 38; 65.5%) or combined with doxorubicin (*n* = 20; 34.5%). Slightly more than half of the patients (*n* = 30; 51.7%) were treated with low-dose cisplatin ≤ 125 mg/m^2^ BSA. Median concentrations of cisplatin were 100 mg/m^2^ BSA (IQR 95.8–108.0) in the low-dose group and 175 mg/m^2^ BSA (IQR 150–175) in the high-dose group. No complications occurred during HITOC.

### Postoperative data

Postoperative complications were documented in *n* = 19 (32.8%) patients, whereby major complications (Clavien–Dindo ≥IIIb) only occurred in 8 (13.8%) patients. Detailed data on the postoperative course are described in Table 2. Median intensive care unit and hospital stay were 2 (IQR 1–3) and 15 days (IQR 12–23) after CRS + HITOC. One patient (1.7%) died in the hospital due to non-surgical complications.

**Table 2:** Postoperative course

Study population, *n* = 58	
Postoperative complications, *n* (%)	
No	39 (67.2)
Yes/Clavien–Dindo classification	19 (32.8)
I	1 (1.7)
II	6 (10.3)
IIIa	4 (6.9)
IIIb	6 (10.3)
IVa	1 (1.7)
IVb	0 (0)
V	1 (1.7)
Surgical revision, *n* (%)	
No	50 (86.2)
Yes/Clavien–Dindo ≥IIIb	8 (13.8)
Haematothorax	2 (3.4)
Pleural empyema	2 (3.4)
Parenchymal/bronchial fistula	3 (5.2)
Other	1 (1.7)
Extubation in the OR	46 (79.3)
Prolongated ventilation >24 h	2 (3.4)
Respiratory insufficiency	2 (3.4)
Prolongated parenchymal fistula (air leak >7 days)	3 (5.2)
Postoperative new atrial fibrillation	2 (3.4)
Postoperative sepsis	4 (6.9)
Postoperative pulmonary embolism	0 (0)
Postoperative pneumonia	8 (13.8)
Renal insufficiency	4 (6.9)
Temporary dialysis	1 (1.7)
Duration of ICU stay (days), median (IQR)	2 (1–3)
Duration of hospitalization (days), median (IQR)	15 (12–23)
In-hospital mortality	1 (1.7)

ICU: intensive care unit; IQR: interquartile range.

### Induction/adjuvant therapy and follow-up

A total of *n* = 25 patients (43.1%) received additive chemotherapy (Table 3). The median follow-up time between start of primary therapy until end of follow-up was 59.0 (95% CI 42.0–76.0) months. At the end of follow-up, tumour recurrence (*n* = 24/49; 48.9%) or tumour progression (*n* = 7/9; 77.8%) was documented in *n* = 31 (53.4%) patients. Most of the patients (93.5%) had ipsilateral tumour recurrence/progression. Of these patients, *n* = 26 (83.9%) patients received further oncological therapy, 3 (9.7%) patients received no further therapy and in 2 (6.5%) patients it was unknown. At the end of follow-up, *n* = 45 (77.5%) patients were still alive. In addition to 1 in-hospital death, during follow-up, a total of *n* = 12 patients (20.7%) died due to primary tumour disease (*n* = 10; 17.2%) or non-surgical reasons (*n* = 2; 3.5%).

**Table 3:** Additive therapy and follow-up

Study population, *n* = 58	
Induction chemotherapy, *n* (%)	20 (34.5)
Adjuvant chemotherapy	7 (12.1)
Additive chemotherapy	25 (43.1)
Adjuvant radiotherapy	13 (22.4)
Tumour recurrence (R0/1, *n* = 49)	24 (48.9)
Tumour progression (R2, *n* = 9)	7 (77.8)
Location of recurrence/progression (*n* = 31)	
Loco-regional	24 (77.4)
Distant metastasis	6 (19.4)
Both	1 (3.2)
Side of recurrence/progression (*n* = 31)	
Ipsilateral	29 (93.5)
Contralateral	2 (6.5)
Therapy of recurrence/progression (*n* = 31)	
No	3 (9.7)
Unknown	2 (6.5)
Yes/type	26 (83.9)
Surgery	4 (15.4)
Chemotherapy	13 (50.0)
Radiotherapy	7 (26.9)
Best supportive care	1 (3.8)
Other	2 (7.7)
Time between start of primary therapy till follow-up end in months,^a^ median (95% CI)	59 (42–76)
Alive at the end of follow-up	45 (77.5)

a Reverse Kaplan–Meier OS.

CI: confidence interval; OS: overall survival.

### Overall and recurrence/progression-free survival

**Figure 1:**
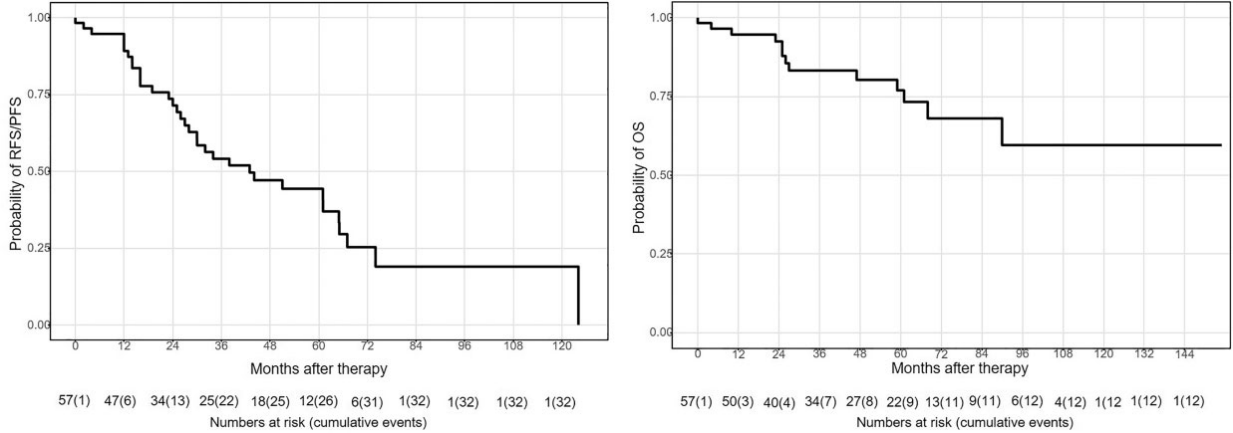
Survival rates for all patients.

**Figure 2:**
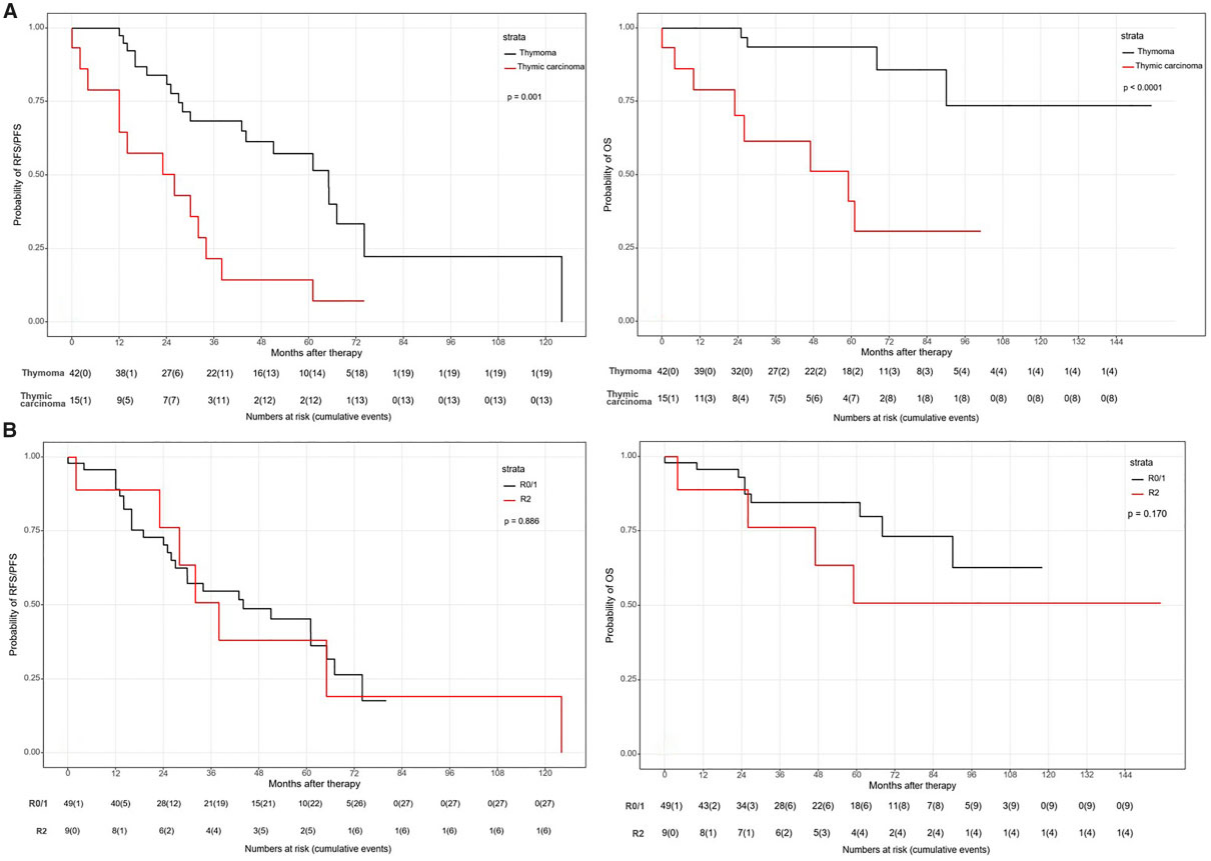
Survival rates depending on histological subtype (**A**) and resection status (**B**).

**Figure 3:**
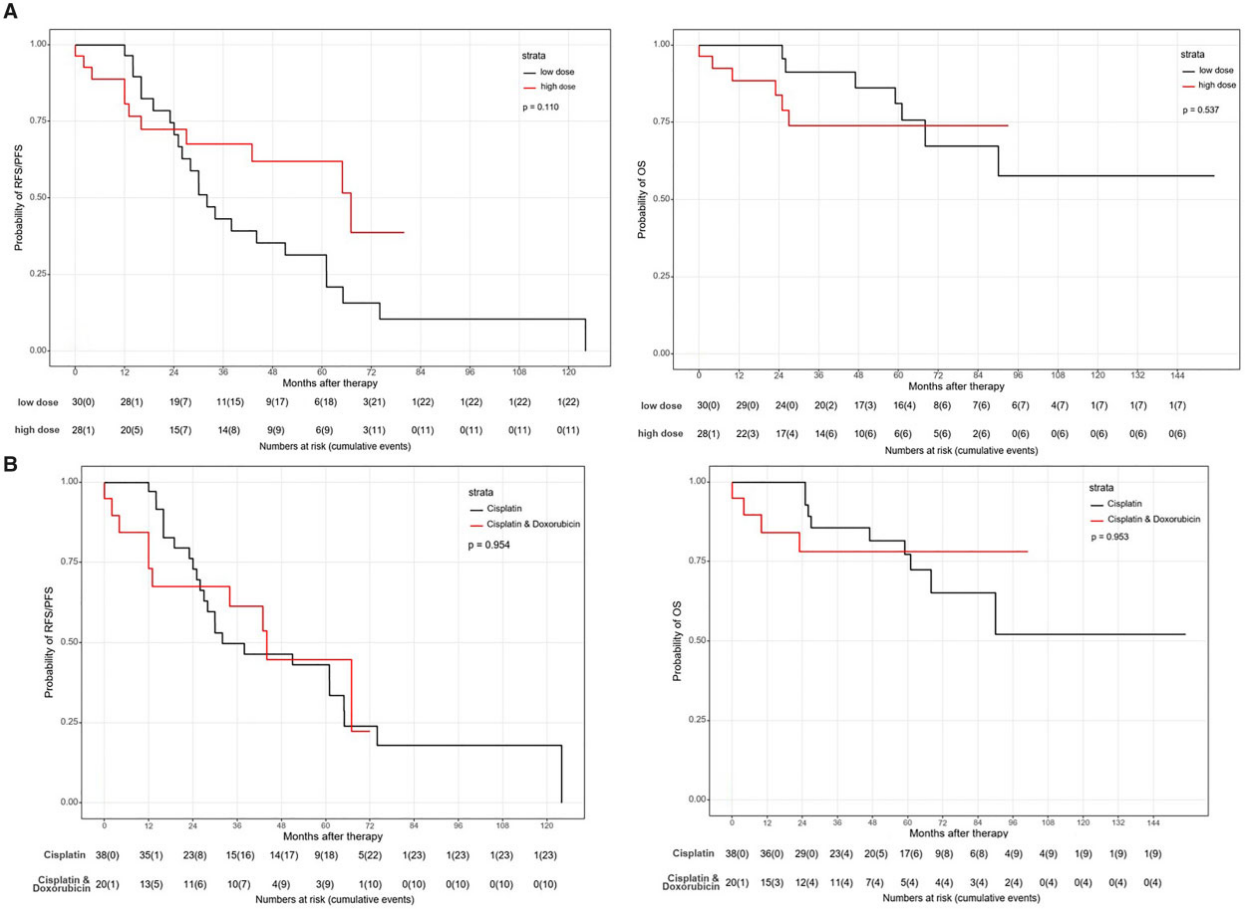
Survival rates depending on cisplatin dosage (**A**) and chemotherapeutical agent (**B**).

**Figure 4:**
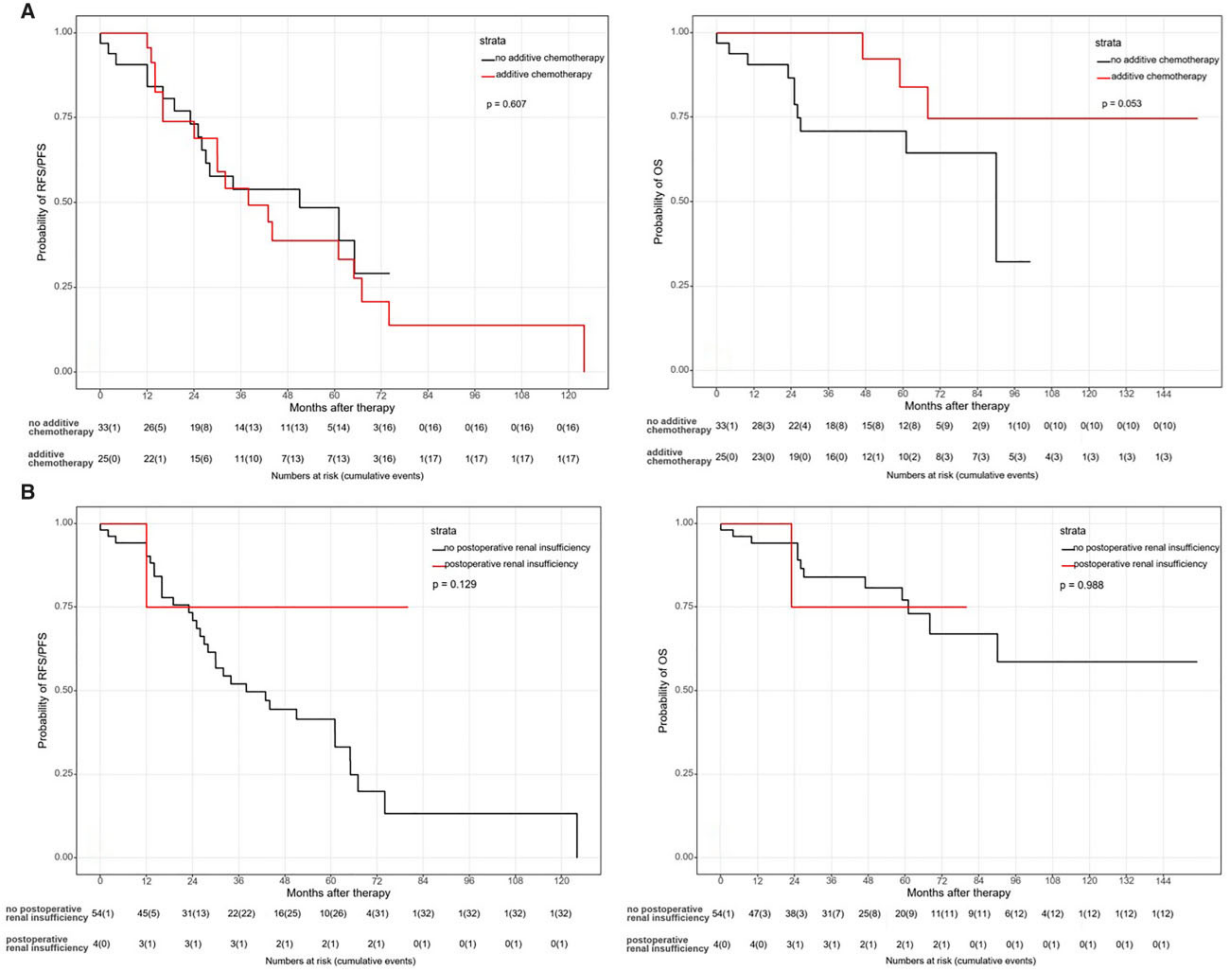
Survival rates depending on additive chemotherapy (**A**) and postoperative renal insufficiency (**B**).

All survival data are presented in Table 4. Overall 1-, 3- and 5-year survival rates were 95%, 83% and 77% (Fig. 1). A significantly better OS was documented in patients with thymoma compared to thymic carcinoma [5-year OS: 94% vs 41%, median survival: 59 months (95% CI 12–106 months); HR 6.77; *P* < 0.001; Fig. 2]. Cisplatin dosage, chemotherapeutical agents, resection status and postoperative renal insufficiency had no significant impact on OS (Figs 2–4). There seems to be a trend for better OS in patients who received additive chemotherapy (*P* = 0.053). In Cox regression analysis, additive chemotherapy showed no effect (*P* = 0.344; HR 0.52; 95% CI 0.13–2.02), but the impact of histological subtype (thymoma) could be confirmed (*P* = 0.003; HR 6.77; 95% CI 1.95–23.46; Table 5).

**Table 4:** Overall survival and recurrence/progression-free survival—survival rates and log-rank tests.

	*n*	*n* events^a^	1-Year survival	3-Year survival	5-Year survival	*P*-Value
OS	58	13	0.95	0.83	0.77	
Cisplatin dose						
Low	30	7	1.00	0.91	0.81	0.537
High	28	6	0.89	0.74	0.74	
Chemotherapeutical agent						
Cisplatin alone	38	9	1.00	0.86	0.77	0.953
Cisplatin + doxorubicin	20	4	0.84	0.78	0.78	
Resection status						
R0/1	49	9	0.96	0.85	0.85	0.170
R2	9	4	0.89	0.76	0.51	
Postoperative renal insufficiency						
No	54	12	0.94	0.84	0.77	0.988
Yes	4	1	1.00	0.75	0.75	
Histological subtype^b^						
Thymoma	42	4	1.00	0.94	0.94	**<0.001**
Thymic carcinoma	15	8	0.79	0.61	0.41	
Additive chemotherapy						
No	33	10	0.91	0.71	0.71	0.053
Yes	25	3	1.00	1.00	0.84	
RFS/PFS	58	34	0.89	0.54	0.44	
Cisplatin dose						
Low	30	23	0.97	0.43	0.31	0.110
High	28	11	0.81	0.68	0.62	
Chemotherapeutical agent						
Cisplatin alone	38	24	0.97	0.46	0.43	0.954
Cisplatin + doxorubicin	20	10	0.73	0.61	0.45	
Resection status						
R0/1	49	27	0.89	0.55	0.45	0.886
R2	9	7	0.89	0.51	0.38	
Postoperative renal insufficiency						
No	54	33	0.90	0.52	0.42	0.129
Yes	4	1	1.00	0.75	0.75	
Histological subtype^b^						
Thymoma	42	20	0.97	0.68	0.57	**0.001**
Thymic carcinoma	15	13	0.65	0.22	0.14	
Additive chemotherapy						
No	33	16	0.84	0.54	0.49	0.607
Yes	25	18	0.96	0.54	0.39	

a In OS analyses, events are defined as deaths from any cause. In RFS/PFS analyses, events are defined as 1st tumour recurrence or progression or death from any cause.

b One patient was excluded due to atypical carcinoid of the thymus.

OS: overall survival; PFS: progression-free survival; RFS: recurrence-free survival.

Bold represents Statistical significant.

**Table 5:** Cox regression analyses for overall survival and recurrence-free survival/progression-free survival

	HR	95% CI		*P*-Value
OS				
Histological subtype (reference = thymoma)	6.77	1.95	23.46	**0.003**
Additive chemotherapy (reference = no)	0.52	0.13	2.02	0.344
RFS/PFS^a^				
Cisplatin dosage (reference = low dosage)	0.64	0.22	1.85	0.409
Chemotherapeutical agent (reference = cisplatin)	2.02	0.71	5.70	0.186
Resection status (reference = R0/1)	0.79	0.30	2.12	0.640
Histological subtype (reference = thymoma)	3.34	1.39	8.02	**0.007**
Additive chemotherapy (reference = no)	2.11	0.93	4.76	0.073

In the Cox regression analyses, 1 patient was excluded due to atypical carcinoid of the thymus. Moreover, a limitation of number of parameters in the multivariable analyses were necessary, only variables with *P*-values <0.1 in univariable analyses were included in the Cox regression.

a The covariate time between initial diagnosis and beginning of primary therapy was included the statistical model.

CI: confidence interval; HR: hazard ratio; OS: overall survival; PFS: progression-free survival; RFS: recurrence-free survival.

Bold represents Statistical significant.

Overall, 1-, 3- and 5-year RFS/PFS were 89%, 54% and 44% and median disease-free interval was 43 months (95% CI 20–66; Fig. 1). With regard to RFS/PFS, only the histological subtype showed a significant impact [thymoma: median = 65 months (95% CI 49–81) vs thymic carcinoma: median = 26 (95% CI 4–48); HR 3.34; *P* < 0.001] with a HR of 3.34 (95% CI 1.39–8.02). The other examined clinical parameters had no impact on RFS/PFS (Figs 2–4), which was confirmed in the Cox regression analysis.

## DISCUSSION

This sub-analysis of the German HITOC study confirmed that encouraging survival rates can be achieved by CRS + HITOC in patients with pleural metastatic TET. In particular, especially patients with thymoma seem to benefit from this multimodal therapy concept, as they achieved an excellent 5-year OS rate of 94%. Furthermore, the 5-year disease-free survival rate of 57% confirms the effective local tumour control by this intrathoracic procedure. Even in patients with incomplete tumour resection (R2), a 5-year OS of 51% could be achieved, which in turn would justify the indication for surgery and possibly tumour debulking at this pleural metastatic stage. Our surgical approach was combined with induction/adjuvant chemotherapy in 43% of patients, which might also have improved survival, even though this factor did not reach statistical significance.

The indication for CRS + HITOC in stage IVa TET should always take the histological subtype according to WHO into account, as it is closely related to stage distribution and survival rates [17, 21, 22]. Respectively, in our analysis, only the histological subtype (thymoma vs thymic carcinoma) could be identified as a significant factor influencing prolonged survival (HR RFS/PFS 3.34 and OS 6.77). Surgery should be even more strictly indicated in patients with stage IVa thymic carcinoma [23]. Maximum tumour resection within the framework of a multimodal treatment concept with chemotherapy and radiotherapy has a deciding influence on survival [24]. Our survival rates of patients with thymic carcinoma stage IVa were promising (5-year OS 41%) compared to the literature (5-year OS 0%), but further investigations are absolutely necessary [9].

Although complete surgical resection has been identified as the prognostically decisive measure for survival in resectable stages I–III, surgical procedures for pleural tumour involvement are still controversially discussed [2, 3]. Several retrospective analyses have demonstrated survival rates of ∼90% at 5 years in both synchronous and metachronous pleural metastases [7, 25]. For both indications, a multimodal therapy should be carefully evaluated. CRS should preferably be performed by lung-preserving procedures (P/D, eP/D), but reports of EPP are also available [26, 27]. In our study group, EPP was performed only in 2 patients if lung-sparing resection was not achievable due to extensive tumour invasion into lung parenchyma. The indication for EPP should be reviewed with careful consideration. Repeated resections in case of pleural or pulmonary recurrence also seem to improve the prognosis [28].

There is only a manageable number of studies in the literature that have investigated surgical tumour resection including HITOC in patients with pleural metastatic TET [1, 9–12]. The safety and feasibility of CRS + HITOC has already been confirmed in numerous studies, while at the same time encouraging data on survival have already emerged [14]. As only available guideline, the European Society for Medical Oncology guidelines recommend that surgery should be offered to patients for whom complete tumour resection is deemed achievable. Hyperthermic intrapleural chemotherapy, as well as EPP, may be discussed in case of TET in stage IVa [6]. Further recommendations in international guidelines regarding surgical resection or the application of HITOC are still lacking [29].

Nevertheless, additional local, intrathoracic procedures have been a topic of discussion again for some years, especially in MPM and TET [30]. Prospective, randomized studies are not available. Therefore, the existing retrospective studies must be considered. For 27 out of 58 patients (47%) of our study, no progression or recurrence was documented during a median follow-up period of 59 months. In cases of pleural progression/recurrence (31/58 patients), it was ipsilateral in 94% of patients. Additional therapy was performed in 24 patients (77%), which might also be an explanation for the prolonged survival despite progression or recurrence.

Unfortunately, the effects of additional HITOC with cisplatin or cisplatin/doxorubicin could not be concretely assessed in the literature as well as in this study due to the limited number of cases. Our data could not show any significant influence of a higher cisplatin dose or an additional administration of doxorubicin. A higher rate of clinically relevant complications in these patients was already excluded from the overall collective [13]. Thus, the question of the optimal dosage or chemotherapeutic agent in patients with TET cannot be answered yet by our study. In our view, however, all local therapies, radical surgical resection and intracavitary chemotherapy can be considered in these patients with pleural metastatic TET.

### Limitations

The study has limitations due to its retrospective nature and its analysis of patients after CRS + HITOC, who were treated in 4 German high-volume departments for thoracic surgery. Likewise, in the absence of a comparison group, we are unable to provide results regarding specific HITOC-associated effects on survival rates. No data on lymph-node dissection were collected, which might have an influence on stage distribution and prognosis. Despite regular follow-up tumour assessment, tumour recurrence or progression might be undetected.

## CONCLUSIONS

Patients with pleural metastatic thymoma showed promising 5-year disease-free and OS rates of 57% and 94% after CRS + HITOC, which justifies this multimodal approach including intracavitary chemotherapy for additional local tumour control. Survival rates after 5 years in patients with thymic carcinoma stage IVa were significantly worse, but encouraging with 14% PFS/RFS and 41% OS. Further data are needed to support the effects of additional HITOC.

## Data Availability

The data underlying this article will be shared on reasonable request to the corresponding author.

## Notes

Presented at the Annual Meeting of the German Society of Thoracic Surgery, Essen, German, 13 September 2022.

## ABBREVIATIONS

BSA: Body surface area
CRS: Cytoreductive surgery
eP/D: Extended pleurectomy/decortication
EPP: Extrapleural pneumonectomy
HITOC: Hyperthermic intrathoracic chemotherapy
IQR: Interquartile range
MPM: Malignant pleural mesothelioma
P/D: Pleurectomy/decortication
SD: Standard deviation
TET: Thymic epithelial tumours
WHO: World Health Organization
